# New insights in the role of *Candida* biofilm in the pathogenesis of recurrent vulvovaginal candidiasis: a prospective clinical study

**DOI:** 10.3389/fmicb.2025.1566171

**Published:** 2025-03-27

**Authors:** Marta Díaz-Navarro, Álvaro Irigoyen-von-Sierakowski, Imani Delcán, Ariadna Monte, María Palomo, Pilar Escribano, Jesús Guinea, Almudena Burillo, Alicia Galar, Patricia Muñoz, María Guembe

**Affiliations:** ^1^Department of Clinical Microbiology and Infectious Diseases, Hospital General Universitario Gregorio Marañón, Madrid, Spain; ^2^Instituto de Investigación Sanitaria Gregorio Marañón, Madrid, Spain; ^3^School of Biology, Universidad Complutense de Madrid, Madrid, Spain; ^4^School of Medicine, Universidad Complutense de Madrid, Madrid, Spain; ^5^CIBER Enfermedades Respiratorias-CIBERES (CB06/06/0058), Madrid, Spain

**Keywords:** biofilm, recurrent vulvovaginal candidiasis, pathogenesis, biomass, metabolic activity

## Abstract

**Background:**

Despite the pathogenesis of vulvovaginal candidiasis (VVC) is multifactorial, this study aimed to assess whether phenotypic characteristics, such as biofilm production and quality, along with clinical symptoms, are associated with recurrent VVC (RVVC).

**Methods:**

Over 1 year (Oct 2021–Oct 2022), we prospectively included 271 patients ≥18 years who attended our institution, had *Candida* spp. isolated in vaginal swabs, and provided informed consent. Patients were followed for 1 year. *Candida* spp. isolates were tested by the following techniques: crystal violet (CV) for biomass quantification, XTT for metabolic activity quantification, and microscopy for biofilm area quantification. Clinical and microbiological data were also collected.

**Results:**

Overall, 55 (20.3%) patients experienced at least one recurrence, with 19 (7.0%) meeting the criteria for RVVC (≥3 episodes/year), with 65 episodes in total. Demographic and clinical characteristics were similar in both study groups. Most isolates were *C. albicans* (90.0%). Median (interquartile, [IQR]) absorbance values for CV and XTT in 18/19 RVVC and 238/252 non-RVVC isolates were as follows: CV, 1.850 (1.578–2.156) vs. 1.426 (1.081–1.823), *p* = 0.005; XTT, 0.184 (0.116–0.293) vs. 0.228 (0.147–0.331), *p* = 0.253. Median (IQR) biofilm occupation area percentage in 16/19 RVVC and 16/252 non-RVVC isolates was, respectively: 13.15 (8.54–16.9) and 10.73 (5.88–17.73), *p* = 0.710.

**Conclusion:**

RVVC was associated to high biomass production. Additionally, RVVC clinical isolates exhibited a tendency toward lower metabolic activity, which may contribute to treatment failure.

## Introduction

Vulvovaginal Candidiasis (VVC) affects about 75% of women at least once in their lifetime and is characterized by *Candida* spp. overgrowth, often associated with non-specific bacterial vaginosis symptoms (Arechavala et al., 2021; Kalia et al., 2020). Recurrent vulvovaginal candidiasis (RVVC) affects about 4–9% of women and is defined as three or more symptomatic episodes of VVC per year, with at least two confirmed by microscopy or culture (Lines et al., 2020; Workowski et al., 2021; Yazdy et al., 2024; Neal and Martens, 2022). The dysbiosis caused by an imbalance between *Candida* spp. and vaginal microbiota is affected by several factors, including prolonged antibiotic usage, childbearing age, and use of contraceptives (Arechavala et al., 2021; Kalia et al., 2020; Neal and Martens, 2022; Brown et al., 2022; Díaz-Navarro et al., 2023; McKloud et al., 2021; Rodríguez-Cerdeira et al., 2020; Tits et al., 2020).

In addition, several *Candida* virulence factors contribute to its ability to infect the vaginal epithelium, particularly biofilm formation (Kalia et al., 2020; Mayer et al., 2013; Iliev and Underhill, 2013; Monfredini et al., 2018; Li et al., 2022; Roudbarmohammadi et al., 2016; Gonçalves et al., 2020; Wu et al., 2020). Another important aspect is the local immune response, where both a deficient immune response to *Candida*, allowing fungal proliferation, and an exaggerated inflammatory reaction against *Candida* are implicated in pathogenesis. These mechanisms have been linked to genetic polymorphisms, including NLRP3 gene variants, and alterations in innate immunity. Besides, it has been suggested that a tightly regulated fungus-host-microbiota interplay might exert a protective role against RVVC (Czechowicz et al., 2022; Rosati et al., 2020; Wang et al., 2024).

VVC treatment typically involves topical antifungal agents or single-dose or two-dose oral antifungal therapy. However, suppressive therapy is required in RVVC patients to achieve full symptom resolution (Denning et al., 2018). Moreover, resistance to conventional antifungals is an increasing problem in recent years, particularly in RVVC (Arechavala et al., 2021; Mesquida et al., 2021). The prevalence of RVVC is expected to rise rather than decline in the future (Denning et al., 2018). Therefore, there is an urgent need to search for alternative treatments that improve patient outcomes (Wang et al., 2024).

However, a deeper understanding of the key factors driving RVVC pathogenesis is essential to identify patients at risk and predict treatment success.

Therefore, the aim of our study was to identify phenotypic and clinical characteristics closely related to RVVC.

## Methods

The study was carried out at a tertiary Teaching Hospital in Madrid (Spain).

### Definitions

#### RVVC

≥3 symptomatic episodes of VVC per year, at least 2 of them confirmed by either microscopy or culture (Workowski et al., 2021; Yazdy et al., 2024; Neal and Martens, 2022).

#### Symptoms

Severity of symptoms (asymptomatic, mild, moderate, and severe) was assessed based on the presence of one or more of the following: vulvar erythema, vaginal itching, leukorrhea, vulvar lichenification.

### Design

Prospective study based on the analysis of *Candida* spp. clinical isolates isolated from vaginal exudate samples of patients with one or more episodes of VVC who attended the Sexually Transmitted Diseases consultation of the Clinical Microbiology Department of our institution.

Vaginal swabs were cultured in CHROMagar™ (BioMérieux, Spain) to isolate yeasts, while MacConkey agar (BioMérieux, Spain) was used to recover lactose-positive Gram-negative bacilli. Clinical isolates were then identified using matrix-assisted laser desorption/ionization time-of-flight mass spectrometry (MALDI-TOF MS). Clinical isolates were stored at −70°C for use in *in vitro* studies.

### Follow-up

Over 1 year (Oct 2021-Oct 2022), patients in whom the presence of *Candida* spp. was detected in vaginal culture were enrolled in the study, with an additional 1-year follow-up (Oct 2022-Oct 2023). Since the presence or absence of VVC was unknown at the time of consultation, informed consent was obtained via telephone once a positive culture was detected. Additionally, patients who returned for follow-up and had initially provided consent by telephone were subsequently given written informed consent during their visit.

### Sample collection

*Candida* spp. clinical isolates from enrolled patients were stored in the mycology laboratory for genotyping and antifungal susceptibility studies.

Once patient follow-up was completed, they were classified into two groups: non-RVVC (patient reporting <3 episodes/year) and RVVC (patient reporting ≥3 episodes/year). Classification was primarily based on the symptomatology reported by the patient, since it was not always possible to isolate the clinical strains for each episode by microbiological culture. Current guidelines do not require confirmation by culture (the British guidelines define that, if there is symptomatology, at least 1 or 2 is enough in order to identify the species) (Workowski et al., 2021). Symptom severity was categorized as mild, severe or asymptomatic.

For the analysis of variables in patients with multiple VVC episodes, the first isolated strain was selected.

### Study variables

The first *Candida* spp. isolate from each patient in both study groups was tested for the following characteristics as previously described (24, 25): biomass production using the crystal violet (CV) assay, metabolic activity using the tetrazolium salt (XTT) assay, and biofilm occupation area using confocal laser scanning microscopy (CLSM). Clinical symptoms were also collected.

For the isolation of *E. coli* in the vaginal swabs, 81 out of 271 samples were available for testing, as its isolation in McConkey agar plates was only performed for a limited period.

### *In vitro* biofilm model for biomass and metabolic activity testing

A 24-h mature biofilm *in vitro* model on 96-well plates. Briefly, a 24-h culture of *Candida* spp. was grown in 20 ml of tryptic soy broth (TSB) at 37°C for 24 h under shaking. The pellets were centrifuged three times, washed with phosphate-buffered saline (PBS), and adjusted to 10^8^ cfu/ml in TSB. Suspensions (100 μl) were inoculated into 96-well plates and incubated overnight at 37°C. The wells were then washed three times with PBS before staining. All assays were performed in triplicate.

For the CV assay, wells were fixed with 125 μl of 99% methanol for 15–20 min at room temperature. Methanol was then removed, and 125 μl of CV was added for 10–15 min at room temperature. After staining, wells were washed with sterile water, and 125 μl of 30% acetic acid was added at room temperature for 10–15 min. Finally, the solubilized CV solution was transferred to a new plate, and absorbance at 550 nm was measured using a spectrophotometer. Results were expressed as the median (interquartile range, [IQR]) of three replicates of biomass absorbance, corrected for the negative control (Høiby et al., 2015; Coenye and Nelis, 2010).

For the XTT assay, 100 μl of XTT (with menadione 1:1000) was added to each well and incubated for 3 h in the dark. Absorbance at 492 nm was measured using a spectrophotometer. Results were expressed as the median (IQR) of three replicates of metabolic activity absorbance value relative to the negative control.

The total number of clinical isolates to be tested was 256/275 (18/19 in RVVC group and 238/252 in non-RVVC group).

### Biofilm occupation area by confocal laser scanning microscopy (CLSM)

For these experiments, 16 clinical isolates from the RVVC group were included (3 out of 19 were not available), including 11 *C. albicans*, 3 *C. glabrata*, and 2 *C. krusei* isolates. Additionally, 16 clinical isolates from the non-RVVC group were arbitrarily selected to match the same species distribution. *In vitro* biofilm models of 24-well plates were used. Biofilms were formed on glass slides (Labolan, Spain) previously coated with poly-L-lysine for 24 h at 37°C. Then, 300 μl of 10^8^ cfu/ml fungal suspension was added to the coated slides placed in the wells, followed by incubation for 24 h at 37°C. Slides were then washed three times with PBS, and 300 μl of formaldehyde was added to each well. The slides were observed under a 20× objective (Leica Geosystems AG, Heerbrugg, Switzerland), and images were processed using FIJI/ImageJ software (National Institute of Health, Bethesda, MD, USA). The median (IQR) percentage of area occupied by *Candida* spp. from the three different surfaces of the slides was calculated (Olson et al., 2018).

In addition, one *C. parapsilosis* strain (arbitrarily selected) and the only *C. orthopsilosis* strain were included for a qualitative assessment of the biofilm structure, allowing for comparison with the biofilms of other species.

### Statistical analysis

Qualitative clinical variables were expressed as counts and percentages and compared using the chi-square test. Quantitative clinical variables were expressed as mean (standard deviation, [SD]) or median (IQR) and compared using the median test.

For the comparison of variables involving 3 or more groups, either the Kruskal-Wallis test or ANOVA was used, depending on data distribution.

Statistical significance was set at *p* < 0.05. All tests were performed using SPSS Statistics for Windows, v.21.0 (IBM Corp, Armonk, New York, USA).

## Results

A total of 271 patients were included in the study, 55 (20.3%) of whom experienced at least one recurrence (≥1 episode/year). Among them, 19 (7.0%) met criteria for RVVC (≥3 episodes/year), accounting for a total of 65 episodes. Particularly, 12 patients had 3 episodes, 6 patients had 4 episodes, and 1 patient had 5 episodes (Table 1). The median (IQR) age of patients was 34.00 (26.00–45.00) years, with no significant differences between study groups (Table 1). Overall, 27.7% of patients had at least one vaginal isolation of *Candida* spp. in the previous year, with a mean (SD) of 1.60 (1.04) episodes, most with no apparent association with specific factors (67.2%). Only 7.7 and 7.4% of previous episodes were related to the menstrual cycle and sexual activity, respectively, and only 0.7% antimicrobial use (Table 1).

**Table 1:** Clinical and microbiological characteristics of the participating patients.

Characteristic, *N* (%)	Group		Overall (*N* = 271)	*p*
	RVVC, *N* = 19	Non-RVVC, *N* = 252		
Median (IQR) age, years	42.00 (27.00–54.00)	33.00 (26.00–44.00)	34.00 (26.00–45.00)	0.079
*Candida* spp. isolation in the previous year	6 (31.6)	69 (27.4)	75 (27.7)	0.791
Mean (SD) of previous episodes	1.67 (1.03)	1.59 (1.05)	1.60 (1.04)	0.871
Previous episodes related to				0.946
No apparent relationship	14 (73.7)	168 (66.7)	182 (67.2)	
Menstrual cycle	2 (10.5)	19 (7.5)	21 (7.7)	
Use of antimicrobials	0 (0.0)	2 (0.8)	2 (0.7)	
Sexual activity	2 (10.5)	18 (7.1)	20 (7.4)	
Previous use of antifungals for VVC	5 (26.3)	33 (13.1)	38 (14.0)	0.160
No. of recurrent episodes		NA		NA
3	12 (63.2)		12 (21.8)	
4	6 (36.1)		6 (10.9)	
5	1 (5.3)		1 (1.8)	
Mean (SD) no. recurrent episodes	3.42 (0.607)	1.97 (0.167)	2.47 (0.79)	**<0.001**
Site of attendance				**0.002**
Outpatient	10 (52.6)	148 (58.7)	159 (58.7)	0.387
Genecology/obstetrics	1 (5.3)	65 (25.8)	66 (24.4)	**0.029**
Microbiology/infectious diseases	4 (21.1)	25 (9.9)	29 (10.7)	0.311
Dermatology	4 (21.1)	13 (5.2)	17 (6.3)	**0.023**
Level of symptomatology^a^				0.258
Mild	6 (31.6)	122 (48.4)	128 (47.2)	
Asymptomatic	3 (15.8)	42 (16.7)	45 (16.6)	
Moderate	6 (31.6)	37 (14.7)	42 (15.5)	
Unknown	5 (26.3)	51 (20.2)	56 (20.7)	
Symptoms				
Vulvar erythema/vaginal itching	12 (63.2)	158 (62.7)	170 (62.7)	0.739
Leucorrhea	11 (57.9)	137 (54.4)	148 (54.6)	0.557
Vulvar lichenification	0 (0.0)	5 (2.0)	5 (1.8)	1.000
*Escherichia coli* co-isolation^*^	0/0 (0.0)	15/81 (18.5)	15/81 (18.5)	0.662

RVVC, recurrent vulvovaginal candidiasis; IQR, interquartile range; SD, standard deviation. Values in bold correspond to statistical significance.

a Level of symptomatology was based on the presence of one or more of the following: vulvar erythema, vaginal itching, leucorrhoea, vulvar lichenification.

* The total available number of samples to be tested for *E. coli* presence was 81/271.

Despite not being statistically significant, patients with RVVC had a higher prior use of antifungals than those with non-RVVC (26.3% vs. 13.1%, *p* = 0.160).

Distribution of *Candida* species in each study group was as follows: RVVC: *C. albicans,* 73.7%; *C. glabrata,* 15.8%; and *C. krusei,* 10.5%; non-RVVC: *C. albicans,* 91.3%; *C. glabrata,* 5.2%; *C. parapsilosis,* 1.6%; *C. krusei*, 1.6%, and *C. orthopsilosis,* 0.4%. A significantly higher prevalence of *C. albicans* was observed in the non-RVVC group compared to the RVVC group (91.3% vs. 73.3%, *p* = 0.029). Moreover, patients with non-RVVC were more frequently attended at Gynecology/Obstetrics Departments (25.8% vs. 5.3%, p = 0.029), whereas patients with RVVC were more frequently seen at the Dermatology Department (21.1% vs. 5.2%, *p* = 0.023) (Table 1).

Regarding symptom severity, most patients presented with mild disease (47.2%), followed by asymptomatic cases (16.6%) and those with moderate disease (15.1%), with no statistically significant differences between groups (*p* = 0.258). The most frequently reported symptoms were vulvar erythema and/or vaginal itching (62.7%) and leucorrhea (54.6%) (Table 1).

Although *E. coli* presence in vaginal swabs could only be analyzed in 81 out of 271 patients, it was exclusively found in the non-RVVC group (18.5%).

### Biomass and metabolic activity production

**Figure 1:**
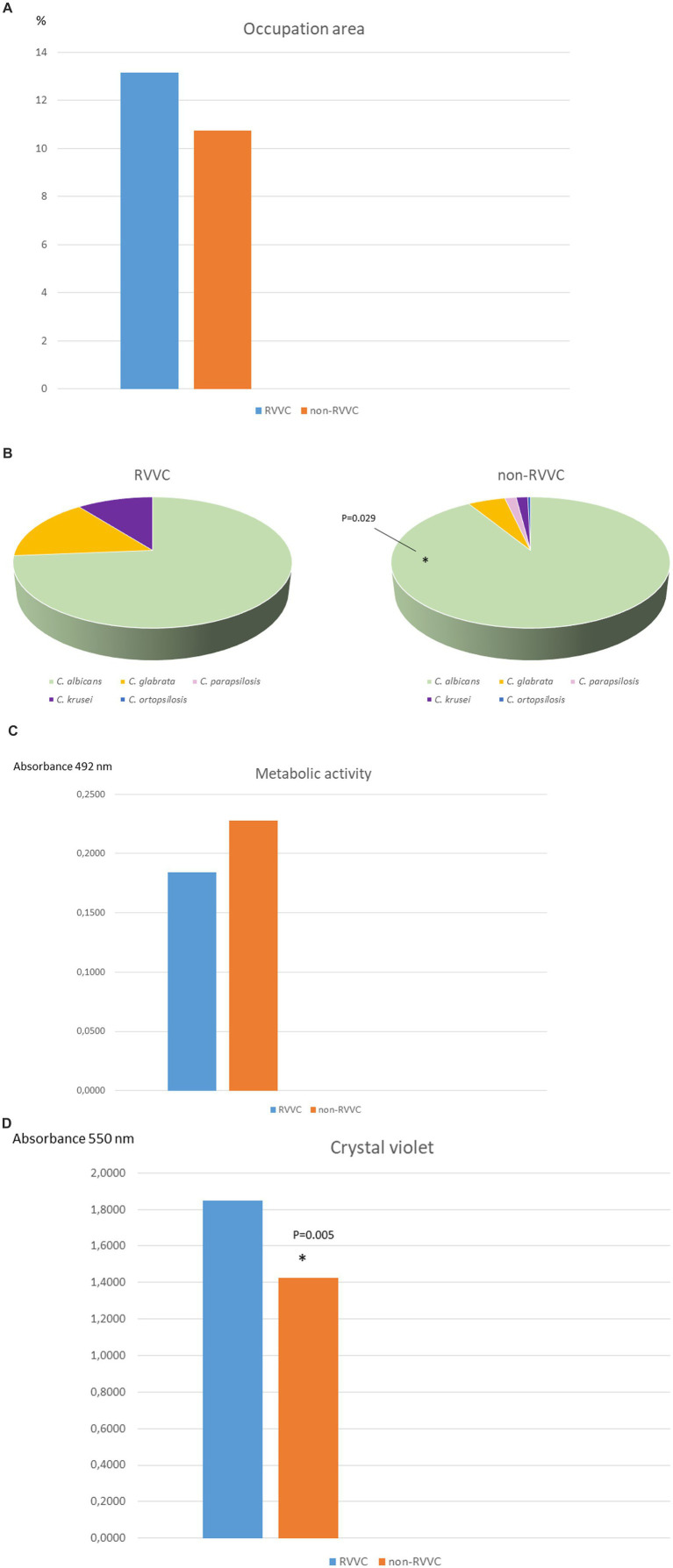
Description of clinical isolates in both study groups. **(A)** *Candida* spp. distribution according to species. * statistical significance *p* = 0.029. **(B)** Median crystal violet absorbance. * Statistical significance *p* = 0.005. The total available number of clinical isolates to be tested was 256/275 (18/19 in RVVC group and 238/252 in non-RVVC group). **(C)** Median metabolic activity absorbance (XTT). NS, non-significant. The total available number of clinical isolates to be tested was 256/275 (18/19 in RVVC group and 238/252 in non-RVVC group). **(D)** Median percentage of biofilm occupation area. NS, non-significant. The total number of selected and available clinical isolates tested were 32 (16 from RVVC group and 16 from non-RVVC).

Analysis was performed on 18/19 RVVC and 238/252 non-RVVC clinical isolates. Median (IQR) CV absorbance was significantly higher in the RVVC group (1.850 [1.578–2.156] vs. 1.426 [1.081–1.823], *p* = 0.005). Despite it did not reach statistical significance, median (IQR) XTT absorbance was slightly higher in the non-RVVC group (0.228 [0.147–0.331] vs. 0.184 [0.116–0.293], *p* = 0.253) (Figure 1).

### Biofilm occupation area by CLSM

**Figure 2:**
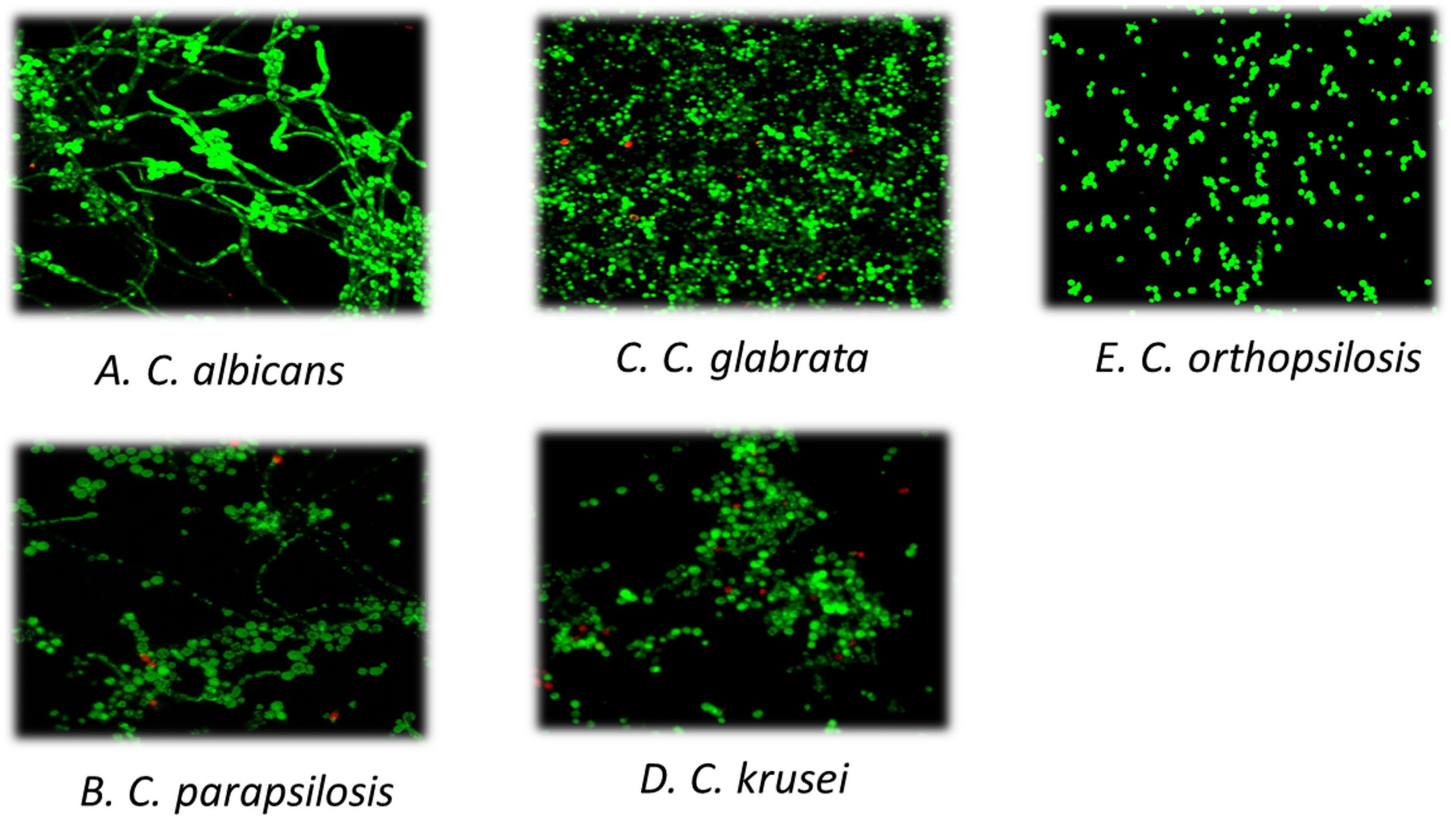
Biofilm structure according to species. We selected a representative image of each *Candida* species. Biofilm formation was performed on 12 mm circular glass slides pre-treated with Poly-L-lysine (1:100) for 24 h at 37°C and observed using confocal laser scanning microscopy (CLSM, Leica Geosystems AG, Heerbrugg, Switzerland) and the APO 63X glycerol immersion objective. Biofilm images (280×280 μm) were captured from three different areas of the slide in triplicate and then processed using the FIJI/ImageJ software (National Institute of Health, Bethesda, MD). The percentage of area occupied by yeasts and hyphae was calculated for each sample. **(A)** *Candida albicans*; **(B)** *Candida parapsilosis*; **(C)** *Candida glabrata*; **(D)** *Candida krusei*; **(E)** *Candida orthopsilosis*.

Analysis was performed in 16/19 RVVC and 16/252 non-RVVC isolates. Median (IQR) percentage of biofilm occupation area was similar between both study groups: 13.15 (8.54–16.49) and 10.73 (5.88–17.73) in RVVC and non-RVVC, respectively (*p* = 0.710). In contrast, when biofilm morphology and structure were analysed according to *Candida* species, we *C. albicans* showed the highest hyphal formation, whereas *C. glabrata* was the one displaying higher density and extracellular matrix (Figure 2). Although only one episode of VVC caused by *C. orthopsilosis* was collected, it showed the lowest biofilm production (Figure 2E).

## Discussion

Considering that RVVC is common condition among women and significantly impacts their sexual health, fertility and reproductive capacity, it is important to investigate the factors contributing to its occurrence to identify effective solutions (Yazdy et al., 2024). Various predisposition factors act as molecular drivers that lead to dysbiosis of the normal vaginal microbiota. Therefore, understanding the molecular mechanisms underlying the virulence pathways driven by these factors is crucial for identifying potential drug targets (David and Solomon, 2023).

In the present study, 20.3% of patients experienced at least one recurrence, and 7.0% were classified as RVVC, having ≥3 episodes/year. Our findings indicate that *Candida* species isolated from women with RVVC had higher biomass and lower metabolic activity. This suggests that sessile cells not only form thicker and denser biofilms but also avoid antifungal action without being fully eradicated. In their dormant state, these cells can persist and later re-infect the vaginal site under conditions of dysbiosis that favor regrowth. In RVVC, dormancy of *Candida* species plays a crucial role in the antifungal treatment failure and subsequent reinfection. Dormant fungal cells, such as persister cells and biofilm-associated *Candida*, exhibit a slowed metabolic state that reduces their susceptibility to antifungal treatments, which primarily target actively growing cells. This resistance mechanism allows *Candida* to survive treatment and later reactivate, leading to reinfection. Additionally, biofilms provide a protective environment that shields the fungus from antifungal agents and immune responses, further contributing to therapeutic failure. As a result, even after apparent resolution, *Candida* can persist in the vaginal mucosa or recolonize from the gut or sexual partners, leading to recurrent episodes despite treatment (Muzny and Schwebke, 2015).

It was also observed that most patients with non-RVVC were diagnosed in the Gynecology/Obstetrics department, while patients with RVVC were mainly diagnosed and followed up in the Dermatology department. This suggests that non-RVVC cases were often incidentally detected during routine pregnancy exams, whereas more complicated, symptomatic and recurrent cases were intentionally monitored by dermatologists. This was corroborated by the higher proportion of moderate-to-severe episodes in the RVVC group (31.6%) compared to the non-RVVC group (14.7%), although this difference was not statistically significant (*p* = 0.258).

Another interesting finding in our study was that biofilm morphology varied depending on the *Candida* species, being *C. albicans* the one that exhibited the highest hyphal formation and *C. glabrata* the one with the lowest hyphae formation, consistent with previous studies (Olson et al., 2018; Ferreira et al., 2009; Pierce et al., 2017). However, these morphological differences were not observed according to biofilm occupation area, as median (IQR) percentage of occupation was similar between RVVC and non-RVVC groups (13.15 [8.54–16.49] vs. 10.73 [5.88–17.73], *p* = 0.710).

Regarding the simultaneous presence of *E. coli* in vaginal cultures, we observed that *E. coli* was only present in non-RVVC patients. This finding warrants further investigation, as it suggests that the simultaneous presence of *E. coli* may protect against recurrence, as we previously demonstrated (Díaz-Navarro et al., 2023).

Moreover, it is important to highlight the need for further research into potential therapeutic strategies for VVC, particularly those targeting probiotics and vaginal microbiota transplantation as means to restore the balance of dysbiotic vaginal microbiota (Wang et al., 2024).

One of the main limitations of the study is that we were not able to recover all clinical isolates for experimental analysis. However, the sample size was high enough to yield reliable results. In addition, we did not included information on the use intrauterine devices. Thus, future studies should evaluate the association between *Candida* biofilms on IUDs and their potential role as reservoirs for reinfection. Moreover, a more in-depth investigation into the role of genetic and immune factors in the pathogenesis of RVVC is necessary.

## Conclusion

RVVC was associated to high biomass production. Although no statistically significant difference was observed, there was a tendency for RVVC clinical isolates to exhibit lower metabolic activity. This may contribute to treatment failure, as antifungal agents have limited efficacy against metabolically inactive cells. We suggest that quantifying biofilm production in *Candida* spp. clinical isolates causing RVVC could provide a better understanding of treatment failures and improve therapeutic strategies.

## Data Availability

The original contributions presented in the study are included in the article/supplementary material, further inquiries can be directed to the corresponding author.
